# Observations on the Fevers of Jamaica

**Published:** 1853-12

**Authors:** 


*Observations on the Fevers of Jamaica.* By J. MAGRATH, Senior Medical Officer of the Public Hospital, Kingston.

During a residence of thirty-five years in this island, I have never met with a single circumstance that for a moment induced me to believe that the fevers which occasionally prevail here epidemically, or arise sporadically, are ever of a contagious nature.

The intermittent, remittent, and yellow fevers appear to me to be modifications of the same disease, influenced by particular circumstances, and to arise from the same cause, viz., a deleterious principle (generated during th decomposition of certain substances, principally vegetable) the production o which is materially influenced by the degree of heat and moisture present, and by the existing state of the atmospheric and telluric electricity.

In 1819, during the prevalence of the fatal epidemic of yellow fever among the troops at Up Park Camp, it repeatedly happened that, on going to the hospital, I passed over spots of small diameter, from whence a hot moist air seemed to issue. Upon afterward examining these situations, I could not, however, discover any cause to account for the circumstance.

I have known patients to be admitted into hospital who, after having had two distinct febrile paroxysms, have lapsed into yellow fever, and died with black vomit. And again, I have known others who were admitted with the muddy injected eye, and drunken suffused appearance of the countenance, deep-seated pain in the orbits, and other pathognomonic symptoms of yellow fever, lasting for two or three days, have afterward two or more paroxysms before they become convalescent. Cases of this kind are rare, but still sufficient, I think, to establish the connection of yellow with intermittent fever. Sir William Pym attempts to distinguish what he calls Bulam from aggravated miasmatic fever; but every symptom he describes as diagnostic of the one I have repeatedly seen in the other.

Yellow fever has not for several years prevailed as an epidemic in Kingston; but every now and then we receive patients suffering from it into the public hospital, and the most of these cases come from coal ships.

In 1848, the crew of a vessel, that had a few months previously been employed in carrying guano, suffered most severely; and in 1849, the persons on board three coal ships, which, after discharging their cargo, took in some impure ballast, were attacked with this disease in its most virulent form, whilst the other vessels in the harbor were comparatively but little affected. The sick from these ships were placed amongst the other patients in the hospital, but the disease did not spread to them.

Unacclimated individuals of every race are liable to attacks of yellow fever, although I believe the negro will often have remittent fever when the white man would be subject to the more aggravated form. I have known persons of color, natives of the mountain districts, and who had never been off the island, to be attacked on coming to Kingston, and die on the third or fourth day, with black vomit, hæmorrhage, etc.

Yellow fever never attacks the thoroughly acclimated, although persons who have had it once are not invariably exempt from a return of it. I have myself attended more than one patient in two well-marked attacks of the disease; and in 1827, shortly after I engaged in practice in Kingston, I was called to see a gentleman, who died on the third day of fever, and who, I was assured by Dr. Adolphus, deputy inspector of hospitals, and by his family, had, about six months previously, a similar attack with well marked black vomit. Persons have been known to die of remittent fever after a residence of upward of twenty years in the island.

We have lately been receiving into the public hospital several persons in the advanced stage of remittent fever, or suffering from its sequelæ, who contracted the disease at Chagres. In them the spleen and liver are always congested, and the lungs occasionally so. The liver and lungs are occasionally disorganized. The greater number are dropsical, and suffering from sub-acute inflammation of the alimentary canal, accompanied by diarrhœa; in several a marked change is observed to have taken place in the blood.

I have seen a few instances of the algide form of fever mentioned by Dr.

Barclay, and believe that when it occurs a paroxysm has taken place, which, from some cause, does not proceed beyond the cold stage, and in which the patient dies or gradually recovers. In 1823, I was called to see a lady in St. Elizabeth's, who had a severe paroxysm of fever, and whom I found in the hot stage. At daylight next morning she was freer from fever, and feeling comfortable. At 9 o'clock, A. M., she complained of being weak, and as I was in the house I saw her immediately, and found her in a state of collapse, which gradually increased until she became deadly cold, like a person in the last stage of cholera; in this state she remained upward of thirty-six hours, when, by the continued use of stimulants, and keeping her wrapped in blankets, she gradually emerged from it, and recovered without any return of fever. Quinine was not in use in this island at that time.

As these different forms of fever arise from the same cause, their treatment is based on similar principles, modified by the intensity of the disease. The indications appear to be, "to relieve such viscera as become the seat of congestion, or are threatened with it; to overcome the fever, and to effect a counterchange in the state of the blood."

General blood-letting in young and otherwise healthy subjects is frequently most useful, in many epidemics, when the patient is seen within the first few hours of the attack; but the indiscriminate practice of it has done a great deal of mischief, particularly when it has been had recourse to after the first day. Topical bleeding is a most valuable remedy, and can be employed at a later period than general blood-letting. Where there is tenderness of the epigastrium, with vomiting and ardent fever, it should rarely be omitted; sinapisms and blisters are also often useful.

The American practice of giving large doses of calomel and quinine is, I think, an excellent one. It is that which we have for the last few years followed in the public hospital, and which I adopt in private practice. The first dose of calomel (a scruple) will often of itself allay the irritation of the stomach, and enable it to retain the scruple of quinine which is given immediately afterwards. The quinine is then given in ten-grain doses, and the calomel in four-grain doses, every third or fourth hour, until an impression is made on the disease, or the quinine is found to be affecting the system, when the dose of it is diminished. Sometimes only the first dose of calomel is found necessary, in other cases several are required. It generally acts on the bowels, and supersedes the necessity of giving those large doses of purgative medicines that were formerly deemed indispensable.

When it is evident that a change is taking place in the state of the blood, the saline medicines recommended by Dr. Stevens are administered, in conjunction with or without quinine, as may be found advisable; and a perseverance in this treatment has saved the lives of many, who without it would have fallen victims to the disease. When the powers begin to fail, stimulants (particularly brandy) are given in moderate quantities.

Kreosote, hydrocyanic acid, and nitrate of silver, have at times been found useful in arresting the vomiting; but in general the oxide of bismuth is found the most effectual remedy.

Black vomit was at one time believed to consist of vitiated bile; but it is now known to be disorganized blood, mixed with the secretions of the stomach. The bile, found in the gall-bladder of patients who die with this symptom, mixes readily with water; whilst a mixture of black vomit and water is turbid, and always deposits a sediment.

The yellow color of the skin, in most instances, arises from the diffusion of bile in the system, and is then first noticed in the eye. In other cases it has its origin in the decomposition of the blood, and in these is first observed in streaks about the neck. When arising from the latter cause, it indicates a most formidable disease.

When an epidemic breaks out, the removal of the people (when it is practicable) should be one of the first steps taken. The good effects arising from this measure were strongly exemplified in the 60th regiment, a few years ago. It was suffering most severely from fever at Fort Augusta; but, on being removed to the mountains, soon got rid of the disease. Had they remained at the Fort their loss would, I have little doubt, have been enormous. —*London Jour. of Med.*, from *N. Y. Jour. of Med.*

